# Innovative Technologies Changing Cancer Treatment

**DOI:** 10.3390/cancers10060208

**Published:** 2018-06-19

**Authors:** Sara Charmsaz, Maria Prencipe, Maeve Kiely, Graham P. Pidgeon, Denis M. Collins

**Affiliations:** 1 RCSI Surgery, Royal College of Surgeons in Ireland, 31A York Street, Dublin 2, Ireland; saracharmsaz@rcsi.ie; 2 School of Biomolecular and Biomedical Research, UCD Conway Institute of Biomolecular and Biomedical Research, University College Dublin, Belfield, Dublin 4, Ireland; maria.prencipe@ucd.ie; 3 Graduate Entry Medical School, University of Limerick, Limerick, Ireland; maeve.kiely@ul.ie; 4 Trinity Translational Medicine Institute (TTMI), St. James’s Hospital and Trinity College Dublin, Dublin 2, Ireland; PIDGEONG@tcd.ie; 5 Cancer Biotherapeutics, National Institute for Cellular Biotechnology, Dublin City University, Glasnevin, Dublin 9, Ireland

**Keywords:** cancer therapeutics, emerging technologies, nanomedicine, computational imaging, cellular stress, radiosensitizing, exosomes

## Abstract

Conventional therapies for cancer such as chemotherapy and radiotherapy remain a mainstay in treatment, but in many cases a targeted approach is lacking, and patients can be vulnerable to drug resistance. In recent years, novel concepts have been emerging to improve the traditional therapeutic options in cancers with poor survival outcomes. New therapeutic strategies involving areas like energy metabolism and extracellular vesicles along with advances in immunotherapy and nanotechnology are driving the next generation of cancer treatments. The development of fields such as theranostics in nanomedicine is also opening new doors for targeted drug delivery and nano-imaging. Here we discuss the use of innovative technologies presented at the Irish Association for Cancer Research (IACR) Annual Meeting, highlighting examples of where new approaches may lead to promising new treatment options for a range of cancer types.

## 1. Introduction

The transformative impact of technological advances on cancer research featured prominently at the 54th annual meeting of the Irish Association for Cancer Research (IACR). Translational research is advancing its enormous potential at breakneck speed, as mature applied technologies meet with the innovations of multi-disciplinary research groups. High-throughput omics studies (e.g., genomics, proteomics, metabolomics, transcriptomics) utilize new technologies to generate enormous datasets that are being mined with ever-more specificity and value [1]. The potential of routine diagnostic technologies, including magnetic resonance imaging (MRI), computed tomography (CT), positron emission tomography (PET) imaging, and immunohistochemical analysis of tissues is being fully understood through the development of the fields of radiomics and pathomics [2,3]. Nanomedicine is harnessing the power of nanotechnology to improve drug delivery, pharmaceutical properties, imaging, and diagnosis, establishing the area of theranostics [4,5]. The ability to isolate, characterise, and functionally phenotype nanometer-scale extracellular vesicles is opening up new possibilities for the therapeutic and diagnostic use of these intercellular RNA, DNA, and protein carriers [6]. Tissue processing and high-throughput fluorimetry underpins novel tools like BH3 profiling to predict cellular response to chemotherapeutic agents [7]. The concept of sensitizing cancer cells to radiotherapy has been investigated for close to 50 years but new advances are seeing more effective small molecule (oxygen/oxygen mimics), macromolecule (miRNA, siRNA, peptide), and engineered nanomaterial-based radiosensitisers emerge [8]. Here we discuss novel studies presented at the 2018 IACR Annual Meeting, which showcased these cutting-edge technologies and their applications in the cancer research arena.

## 2. Nanomedicine in Cancer Treatment

In recent years, the development of nanomedicine has shown great promise for both advanced imaging and therapeutic capabilities against cancer. Nanomedicine is a form of nanotechnology applied to the biomedical field, in which engineered nanoparticles (NPs) with dimensions of less than 100 nm are used to treat disease, primarily cancer. With respect to traditional cancer therapies, such smart and highly engineered NPs provide advantages in passively or actively targeting drugs, with high solubility, bioavailability, biocompatibility, and multifunctionality. These NPs can be used in multimodal therapies. Several kinds of smart NPs have thus been developed for targeted drug delivery, as multi-target inhibitors, and as nano-imaging tools. Only recently, the combination of both therapy and diagnosis was proposed to operate using the same nanoparticle, thus introducing the concept of theranostics to the field of nanomedicine field [9]. The core concept is thus to have a multi-functional NP able to perform imaging and diagnosis at the site of work, i.e., cancer cells, and also treat them. The therapy would possibly be delivered in a multi-modal approach, for example, combining drug or gene delivery with external stimuli, like hyperthermal, photothermal, or photodynamic (PDT) treatments, thus attacking the cancer cells with different mechanisms. Moreover, high colloidal stability in biological fluids, selective targeting capabilities, and final biodegradation are other features included in these highly engineered theranostics nanoplatforms. In this way, nanomedicine applied with theranostic nanoparticles shows promise and may potentially be able to overcome the common disadvantages of conventional therapies against cancer, i.e., lack of high selectivity and discrimination among healthy and cancer cells, adverse effects towards normal cells, acquired drug resistance, and lack of early diagnosis and methods of molecular imaging.

Prof. Valentina Cauda and her group (Department of Applied Science and Technology (DISAT) of Politecnico di Torino, Turin, Italy) have reported some recent results on smart nanoparticles that are able to successfully visualize and kill cancer cells in vitro. At first, insights on silica nanoparticles with a highly porous structures and tiny nanopores of around 3–4 nm in diameter were given, i.e., mesoporous silica nanoparticles (MSNs). These MSNs can be easily chemically functionalized [10] to show colloidal and anti-thrombogenic behaviour in biological fluids [11], preventing unwanted aggregation or early biodegradation [12], and targeting cancer cells [13]. Moreover, these MSNs are able to incorporate anticancer drugs [14] and/or fluorescent molecule reporters [15] for both drug delivery and molecular imaging at the desired site of interest.

Particular attention was given to a novel and multifunctional nanoparticle based on a dense nanocrystalline metal oxide core (zinc oxide) shielded with a lipidic bilayer able to be highly stable in biological media, even for long-term applications, with the intention of rendering it injectable into the blood stream [16]. Moreover, its therapeutic capabilities were reported to be dependent not on drug delivery but on a stimuli-responsive mechanism. Ultraviolet light stimulation for few seconds (photodynamic therapy—PDT) is able to excite the nanocrystalline particles and generate, intracellularly, highly toxic reactive oxygen species (ROS) [17]. The first cytotoxicity tests on Hela cancer cells showed a cell killing rate of 65–80% when the nanoparticles were stimuli-activated. There was no effect on tumour cell viability following UV stimulation on its own or using the nanoparticles without stimulation.

These results show the promising role of highly engineered and functionalized nanoparticles for cancer treatment and molecular imaging. However, global success in cancer therapies based on such nanomedicine tools can be achieved only with a profound knowledge of tumour biology, markers, and microenvironment.

## 3. Radiomics and Pathomics

Traditional biology generally attempts to molecularly dissect diseases and study them part by part with the idea that the sum of knowledge of the parts will help explain the disease as a whole. This strategy has only rarely been successful when trying to understand the causes and cures for complex diseases. Therefore, a systems-based approach seems a better strategy to study and understand this complexity. Specifically, this approach takes into account a large number of interrelated variables such as gene expression profile, tumour cellular architecture, and microenvironment (seen through histological image features), three-dimensional tissue architecture and vascularization (seen through dynamic contrast-enhanced (DCE) magnetic resonance imaging (MRI)), and metabolic features (seen through magnetic resonance spectroscopy (MRS) or PET), to identify a specific disease phenotype. Prof. Anant Madabhushi (Director of the Center for Computational Imaging and Personalized Diagnostics (CCIPD) at Case Western Reserve University, Cleveland, OH, USA) presented some of the tools that they have developed to integrate and correlate heterogeneous biological data spanning different spatial and temporal scales, modalities, and functionalities. These tools include computerized feature analysis methods for extracting sub-visual attributes for characterizing disease appearance and behaviour on radiographic (radiomics) and digitized pathology images (pathomics). At the IACR meeting, Prof. Madabhushi presented a number of examples of the application of radiomics and pathomics to predict disease outcome, recurrence, progression, and response to therapy which are reported below.

(1) Computational imaging of digital pathology for risk stratification of cancers: Prof. Madabhushi’s group has been developing powerful computerized image-based risk assessment tools for the mining of digital pathology images for outcome prediction and risk stratification of prostate cancers [18], breast cancers [19], and p16+ oropharyngeal cancers [20]. Specifically, Prof Madabhushi’s group showed that image-derived biomarkers from digitized tissue images of oestrogen receptor-positive (ER+) breast cancers were highly correlated with Oncotype DX, a USD $4000 21-gene expression panel for risk stratification of breast cancer [19]. This approach thus has the potential to complement—and in some cases obviate—the need for more expensive molecular-based assays for personalizing treatment strategies.

(2) New radiomic approaches for diagnosis and prognosis of disease on radiologic imaging: Prof. Madabhushi’s group has developed a new image-based descriptor called CoLlAGe (Co-occurrence of Local Anisotropic Gradient orientations) [21] which allows for quantification of entropy associated with pixel-level image gradients. The CoLlAGe descriptor has been successfully used to distinguish between radiation necrosis (a benign effect of radiation therapy) and cancer recurrence from a routine MRI scan. Currently radiologists are unable to tell these two pathologies apart using an MRI scan. As a result, thousands of patients with radiation necrosis have to undergo unnecessary cranial biopsies to confirm absence of cancer.

(3) Fused imaging-molecular predictors of treatment outcome: Prof. Madabhushi presented a new computational image and bioinformatic algorithm [22] to combine disease patterns (as identified through histologic imaging) with proteomic data. This leads to better predictors of disease risk stratification and treatment response. These methods have recently been successfully applied to predicting disease recurrence in the context of men with prostate cancer where the combined imaging and omics predictor was shown to be more accurate compared to just imaging or “omics” alone.

## 4. Enhancing Radiotherapy Treatment

Radiotherapy is a main treatment modality for approximately 50% of cancer patients and second only to surgery in curative ability. However, the biological mechanisms conferring radioresistance in inter- and intra-cell types are poorly understood. The importance of the mitochondria and its links with radiation resistance are topical in the literature. During the last decade, numerous approaches that selectively target cancer cells by virtue of their mitochondrial defects have been shown to exert anti-tumour effects [23]. Hypothetically, the most efficient mitochondrial therapies would be those that affect processes in mitochondria linked to key features of the neoplastic phenotype. Damage to the mitochondria is at the crossroad between normal metabolism and the regulation of cell death, and represents an important direction for the development of new therapies. Those targets that can regulate mitochondria function and metabolism and simultaneously increase sensitivity to induction of apoptosis may be the most effective anti-cancer agents.

Prof. O’Sullivan’s group (Trinity Translational Medicine Institute, Trinity College Dublin) has a particular focus on understanding the role of the mitochondria and energy metabolism in models of oesophageal cancer (OAC) radiation resistance. In Ireland, incidence of OAC has increased by 48% over the past 15 years and the overall cure rate is less than 20% [18]. Consequently, a multi-modal approach to treating this disease involves neoadjuvant treatment (treatment prior to surgery) with either chemotherapy alone or combination chemoradiotherapy (neo-CRT) for locally advanced tumours [24,25]. Unfortunately, only ~30% of patients show a beneficial response with ~70% of patients receiving a toxic treatment with no benefit. These patients also experience a delay to surgery which may significantly affect overall patient survival.

Using an isogenic model of OAC radioresistance, Prof. O’Sullivan’s group has shown that radioresistance is associated with altered mitochondria structure and size. The levels of random mitochondrial mutations and altered metabolic profiles demonstrated metabolic plasticity, efficiently switching between glycolysis and oxidative phosphorylation energy metabolism pathways accompanied by enhanced clonogenic survival [26]. In vivo using patient samples, energy metabolism as measured by the levels of ATP synthase F1 subunit beta (ATP5B) can significantly segregate responders and non-responders to neoadjuvant chemoradiation treatment [26].

Through a gastrointestinal drug discovery programme, Prof. O’Sullivan’s group has identified and patented a novel radiosensitiser with dual anti-metabolic and anti-angiogenic activity. Using an in vivo zebrafish model and human ex vivo OAC explant models, they have shown this small molecule inhibitor can significantly reduce both metabolic and anti-angiogenic activity in real time and in parallel with increasing radiosensitivity in an isogenic model of radioresistance. Importantly, the action of this novel small molecule is also effective under hypoxic conditions. Prof. O’Sullivan’s group is building further pre-clinical data on the use of this novel radiosensitiser in the neoadjuvant treatment setting for gastrointestinal malignancies. 

## 5. RNase-Based Transcriptome Interrogation

Cellular stress responses are essential for tumour suppression in healthy cells. Stress responses mediated by checkpoints in numerous complex signalling pathways allow the cells time to repair the damage induced by a plethora of potentially dangerous events (including dysregulated proliferation and nutrient deprivation) [27]. If the damage cannot be repaired, cells can trigger arrest of the cell cycle or induce cell death mechanisms as a protective measure. Exploitation of these cellular stress responses in cancer therapeutics has the potential to slow down cancer cell growth but also to induce the death of the cancer cell.

The endoplasmic reticulum (ER) is a major site for protein folding and quality control. Tumour cells need to overcome powerful ER stress responses to survive. However, even when it does this, the tumour cell can still remain vulnerable to therapeutic opportunities [28]. Dr. Eric Chevet and his group (Centre de Lutte Contre le Cancer Eugène Marquis, Université de Rennes, Rennes, France) have demonstrated that ER stress and the unfolded protein response (UPR) play an instrumental role in the development of glioblastoma multiforme (GBM). GBM is the most severe form of primary brain cancer, representing more than 15% of all brain tumours [29]. Despite aggressive treatment comprised of surgical resection and radio/chemotherapy, patient survival post-diagnosis of GBM remains short, with a median of 15 months [30].

Dr. Chevet has found that the ER stress sensor IRE1alpha (referred to as IRE1) contributes to GBM progression, impacting both tissue invasion and tumour vascularization. IRE1 is an RNase that signals by catalysing the splicing of mRNA encoding the transcription factor X-box binding protein 1 (XBP1), in addition to regulating the stability of certain miRNAs and mRNAs through a process known as regulated IRE1-dependent decay (RIDD). Somatic mutations in the IRE1 gene have been identified in GBM and other forms of cancer. Taking advantage of the specific signalling outputs of the RNase domain of IRE1 engaged by distinct GBM-related mutations, they defined specific expression signatures that were then investigated in the context of human GBM transcriptomes. This approach allowed them to demonstrate the antagonistic roles of XBP1 mRNA splicing and RIDD on tumour outcomes. As part of this response, they demonstrated that the IRE1/XBP1 axis ensures the secretion of pro-inflammatory chemokines by the tumour cells, thereby promoting the recruitment of tumour-associated macrophages, microglial cells and to some extent, neutrophils. This was demonstrated with cell lines, primary GBM lines, and using in vivo GBM orthotopic mouse models and patient tumour samples [31].

This study provides the first demonstration of a dual role of IRE1 downstream signalling in cancer and opens a new therapeutic window to abrogate tumour progression. This study also indicates that ER stress as well as UPR and IRE1 signalling might represent appealing targets for the development of therapeutics that could in turn be used either alone or as adjuvant to the current treatments.

## 6. Dynamic BH3 Profiling

Apoptosis is one of the main mechanisms of response to cellular stress which function through clearance of dysfunctional cells that pose a risk to the healthy organism [32]. Apoptosis occurs through two major pathways: the extrinsic (death receptor pathway) and the intrinsic (mitochondrial pathway). In the intrinsic apoptotic pathway, a subset of BCL-2 family proteins called “BH3-only” play a key role in promoting apoptosis in response to cellular stress cues [32]. These BH3-only proteins interact with other pro-apoptotic and anti-apoptotic proteins members of the BCL-2 family to decide cell fate.

Dr. Letai’s group (Dana-Farber Cancer Institute, Boston, MA, USA) takes a different approach towards precision medicine, based on the belief that the most important outcome of precision medicine in cancer is to match the right patient to the right drugs. Many studies on precision medicine are exclusively focused on the study of the cancer genome. While genomic approaches have yielded some exciting successes, it must be acknowledged that the vast majority of cancer patients do not derive direct therapeutic benefit from such approaches. Therefore, new avenues must be investigated to improve the current situation [33].

One characteristic that genomic approaches have in common with most (if not all) omic approaches is that they are static studies of the cancer cell. In fact, the first step one takes to initiate most of these studies is to kill the cancer cell. The valuable functional information from the genomic studies can be lost when the cancer cell is reduced to a bag of molecules. The emergent properties of the immense complexity of interactions among macromolecules are poorly predicted by initial condition measurements alone. Therefore, Dr. Letai’s group has focused on making targeted perturbations of living, functioning cancer cells. To do this the patient’s cancer cells are put in direct contact with the therapeutics of interest. Following this perturbation, it is essential to measure a component that rapidly captures key information about the ability of the therapeutic that kill the cancer cells. To achieve this, they have focused on profiling BH3-only proteins. BH3 profiling, as shown by IACR member Dr. Triona Ni Chonghaile (Royal College of Surgeons in Ireland, Dublin, Ireland) [34], can identify how close a cell is to the threshold of apoptosis. When this approach is preceded by a brief ex vivo drug treatment, dynamic BH3 profiling can measure the potential of a drug to push a cell closer to the threshold of apoptosis. Data from Dr. Letai’s group have shown that taking this approach could be an excellent predictor of the efficacy of a specific drug in vivo. Since an exposure of no more than 24 h is required, long -term ex vivo culture of cancer cells is not required, rendering this strategy feasible for almost any primary cancer tissue. Currently Dr. Letai and his group are pursuing this strategy in a wide variety of both solid and liquid tumours.

## 7. Extracellular Vesicles as a Therapeutic Strategy

Prof. Clotilde Thery (The Institut Curie, Paris, France) delivered the annual Irish Cancer Society keynote lecture on the molecular and functional diversity of exosomes and other extracellular vesicles in tumour-immune system cross-talk. Cells secrete different types of extracellular vesicles (EVs) into their environment with distinct properties depending on their intracellular site of origin. Exosomes are a subtype of EVs with a mean diameter lower than 150 nm that are formed inside multivesicular compartments of the endocytic pathway. Exosomes secreted by dendritic cells (DCs) have been shown to bear functional major histocompatibility complex (MHC) class I and class II molecules able to activate cognate T lymphocytes and induce anti-tumour immune responses. These findings motivated the use of DC-derived exosomes in cancer clinical trials although with limited clinical effects. Other EVs also bear functional immune molecules and may thus represent alternative immunotherapy tools, but side-by-side comparison of exosomes and other EVs in terms of biochemical and functional properties must be performed to determine their respective therapeutic values.

Prof. Thery described her recent work optimising the isolation of different subtypes of EVs simultaneously released by live human primary DCs to characterise their protein composition and their abilities to activate T lymphocytes. Specific markers to allow isolation and analysis of EVs were an unmet need. Extensive proteomic analysis identified markers used for exosome identification (MHC, flotillin, HSP70) as general markers of EVs [35]. Within small EVs, Prof. Thery’s group identified exosomal and non-exosomal subpopulations that can be differentiated using CD9, CD63 and CD81. An additional layer of complexity was demonstrated through the findings that the functionality of EV subtypes produced by immature DCs differs from those derived from mature DCs [36]. When produced by IFN-gamma matured DCs, both large and mixed exosomal/non-exosomal small EV populations induce secretion of TH1-cytokines. However, large EVs produced by immature DCs induce secretion of TH2-associated cytokines with the small EVs maintaining the Th1 cytokine profile. Now that the isolation methodologies and functional characterisation are advancing, Prof. Thery emphasised the need to determine the respective roles of exosomes and other EVs in cancer:immune system cross-talk to fully exploit these potentially powerful and versatile biological tools [37]. EV-TRACK (http://evtrack.org) is a valuable resource for EV scientists to ensure the standards of EV isolation and characterisation, while developments in the area can be followed through meetings of the International Society for Extracellular Vesicles (www.isev.org).

## 8. Discussion and Concluding Remarks

It is clear that innovative technologies are being utilized to inform and alter our understanding and treatment of cancer. While all of the technologies discussed at the IACR face challenges and are still developing, many are already impacting patients. Approved clinical stage nanomedicines are exemplified by liposomal irinotecan for metastatic pancreatic cancer and nab-paclitaxel for breast lung and pancreatic cancer [4]. Radiomics approaches have been shown to enable differentiation of cancerous from noncancerous prostate tissue [38]. Multiple radiosensitisers are currently being assessed in the clinic across a breadth of disease types including prostate and cervical cancer as well as glioblastoma [8]. High-throughput transcriptomics such as next-generation RNAseq has become a cornerstone of translational protocols with gene expression profiles generated from RNA transcripts forming the basis of tests like Oncotype DX, used routinely to predict treatment benefit in breast cancer [39]. BH3 and the metabolomic profiling of oesophageal cancers are currently undergoing clinical investigation [NCT03223662]. Dendritic cell exosomes have been the subject of clinical studies in lung, colorectal, and breast cancers [40].

Each plenary session at the 2018 IACR annual meeting featured advanced technology employed directly and indirectly in the research reported. Technology is now essential in the progression of modern cancer treatment, pushing the boundaries of personalized and targeted medicine to improve patient outcomes. Exciting new possibilities will continue to emerge as knowledge, technical expertise, and accessibility converge within multi-disciplinary academic research groups.

## Appendix A

**Table A1:** Contributing speakers.

Speaker	Affiliation
Prof. Valentina Cauda	Politecnico di Torino, Italy
Prof. Anant Madabhushi	Center for Computational Imaging and Personalized Diagnostics, Case Western Reserve University, USA
Prof. Jacintha O’Sullivan	Trinity Translational Medicine Institute, Trinity College Dublin, St. James’s Hospital, Ireland
Dr. Eric Chevet	French Institute of Health and Medical Research, University of Rennes, France
Dr. Anthony Letai	Dana-Farber Cancer Institute, Harvard Medical School, USA
Prof. Clotilde Thery	French Institute of Health and Medical Research, Institut Curie, PSL Research University, France
